# Pamiparib as consolidation treatment after concurrent chemoradiotherapy of limited-stage small cell lung cancer: a single-arm, open-label phase 2 trial

**DOI:** 10.1186/s13014-024-02437-2

**Published:** 2024-04-12

**Authors:** Jiuang Mao, Jianjiao Ni, Li Chu, Xiao Chu, Dayu Xu, Xi Yang, Zhengfei Zhu

**Affiliations:** 1 Department of Radiation Oncology, Fudan University Shanghai Cancer Center, Shanghai, 200032 China; 2 Department of Oncology, Shanghai Medical College, Fudan University, Shanghai, 200032 China; 3 Shanghai Clinical Research Center for Radiation Oncology, Shanghai, 200032 China; 4 Shanghai Key Laboratory of Radiation Oncology, Shanghai, 200032 China; 5 Institute of Thoracic Oncology, Fudan University, 270 Dongan Road, Shanghai, 200032 China

**Keywords:** Local-stage SCLC, PARP inhibition, Pamiparib, Consolidation treatment

## Abstract

**Background:**

Small cell lung cancer (SCLC) is highly invasive with poor prognosis, and its treatment has historically been hindered due to the absence of targetable driver genomic alterations. However, the high genomic instability and replication stress in SCLC have made poly(ADP-ribose) polymerases (PARPs) inhibitors a focus of research. Pamiparib is an orally available PARP1/2 inhibitor with high selectivity, strong PARP trapping activity, and excellent brain penetration. Utilizing pamiparib as consolidation maintenance therapy in limited-stage SCLC holds promise for improving survival outcomes and offering a viable therapeutic approach.

**Methods:**

This single-arm, open-label phase II trial will enroll patients aged 18–75 years with histologically/cytologically confirmed, limited-stage SCLC who have not progressed following definitive platinum-based cCRT and have an ECOG PS of 0 or 1. Patients will be excluded if they have histologically confirmed mixed SCLC or NSCLC, or have undergone previous tumor resection, or can be treated with surgery or stereotactic body radiation therapy/stereotactic ablative radiation therapy. Participants will receive pamiparib 40 mg twice daily every 3 weeks within 2 to 6 weeks after cCRT for up to 1 year or until disease progression according to RECIST v1.1. The primary endpoint is the 1-year progression-free survival (PFS) rate assessed by investigators per RECIST v1.1. Secondary endpoints include PFS, objective response rate, and duration of response assessed by investigators per RECIST 1.1, overall survival, time to distant metastasis, and safety.

**Discussion:**

The study will provide valuable data on the feasibility, safety, and effectiveness of pamiparib as a consolidation therapy after cCRT in patients with LS-SCLC. The correlation between molecular typing or gene expression profile of the disease and curative response will be further explored.

**Trial registration:**

NCT05483543 at clinicaltrials.gov.

## Background

Small-cell lung cancer (SCLC) accounts for 14% of newly diagnosed lung cancer cases and is characterized by its high invasiveness, early metastasis, and poor prognosis. Although the incidence of SCLC has declined in most age groups, the median overall survival (mOS) has shown minimal improvement over the past three decades, remaining stagnant at 7 months [1, 2]. Limited-stage (LS) SCLC is defined as lesions that can be included in a single radiation field. Currently, approximately 30% of SCLC patients are initially diagnosed with LS. The standard treatment is thoracic radiotherapy (DT60-66 Gy/33 Fx, 2 Gy/Fx or DT45 Gy/30 Fx, 1.5 Gy bid) in combination with 4 courses of cisplatin/carboplatin plus etoposide chemotherapy (National Comprehensive Cancer Network®) [3]. Despite the initial high response in the first-line treatment of LS-SCLC, relapse inevitably occurs with only approximately 10% of patients maintaining a disease-free status after 2 years [4]. Multiple maintenance therapies, including immunotherapy, targeted therapy, and chemotherapy-based combination therapy, have been studied, yet their effectiveness remains constrained [5–7]. While some studies have reported marginal enhancements in PFS, the OS data from almost all trials have demonstrated negative results [8, 9]. More effective approaches that could delay disease recurrence and improve outcomes for SCLC patients after the initial treatment are needed.

Poly (ADP-ribose) polymerase (PARP) is a diverse family of enzymes involved in ADP-ribose transfer, with PARP-1 and PARP-2 playing critical roles in the DNA damage response (DDR) [10]. Ovarian and breast cancers with BRCA mutations suffer from deficiencies in the homologous recombination (HR) pathway, making them highly susceptible to the synthetic lethality induced by PARP inhibitors (PARPi). This forms the fundamental basis for their current application in anticancer therapy [11]. Even beyond the HR-related context, PARPi itself acts as a DNA-damaging agent by capturing PARP1, resulting in the accumulation of single-stranded DNA (SSD) and impeding the progression of DNA replication forks, ultimately leading to the formation of DNA double-strand breaks (DSBs) [12].

Preclinical studies and genomic/transcriptomic analyses have revealed the vulnerability of SCLC to PARP inhibitors. Firstly, SCLC cell lines and tumors show significantly higher levels of PARP1 protein and mRNA compared to healthy lung tissue and other types of lung cancer [13]. Secondly, despite SCLC tumors naturally having the ability for homologous recombination (HR), preclinical data still strongly indicate their remarkable responsiveness to PARPi [14, 15]. This sensitivity could be attributed to the distinct biological features of SCLC’s DNA replication stress. Several factors, including the inactivation of tumor suppressor genes p53 and RB1, amplification of the MYC family, ongoing abnormal cell growth and metabolism [16–18], accumulation of reactive oxygen species (ROS) within cancer cells [19, 20], and unconventional protein post-translational modifications [21], collectively define the distinctive biological nature of DNA replication stress in SCLC. Furthermore, PARPi has also been proven to be an effective chemoradiosensitizer, significantly increasing mortality in various tumor models [22, 23]. Clearly, PARPi demonstrates significant potential in treating SCLC, given its primary pharmacological action targeting DNA replication, which is in line with the biological features of SCLC and its sensitivity to radiotherapy and chemotherapy. The alignment between PARPi and the heterogeneous biological characteristics and treatment strategies of SCLC undoubtedly provides strong support for conducting further clinical trials.

Pamiparib is a potent small molecule inhibitor of PARP-1 and PARP-2, showing superior cytotoxicity compared to other PARP inhibitors. Notably, it exhibits exceptional blood-brain barrier (BBB) permeability in mouse models, enabling effective penetration into the central nervous system (CNS) for tumor therapy [24, 25]. Conditional approval has been granted by the China National Medical Products Administration (NMPA) for treating patients with recurrent advanced ovarian cancer carrying germline BRCA mutations after at least two lines of chemotherapy [26]. Promising efficacy with acceptable tolerability has been demonstrated in preclinical studies and clinical trials for a range of solid tumors, including ovarian, breast, and lung cancers [27–30].

Building upon this foundation, we conducted a phase II, single-arm, prospective study to assess the efficacy and safety of pamiparib as a monotherapy maintenance treatment and investigate its mechanism of action in local-staged small cell lung cancer, with the aim of providing improved therapeutic options for patients. Furthermore, this study will facilitate discussions surrounding optimal drug utilization, patient selection criteria, and timing of pamiparib treatment in limited-stage small cell lung cancer.

## Methods and design

### Study design

**Fig. 1:**
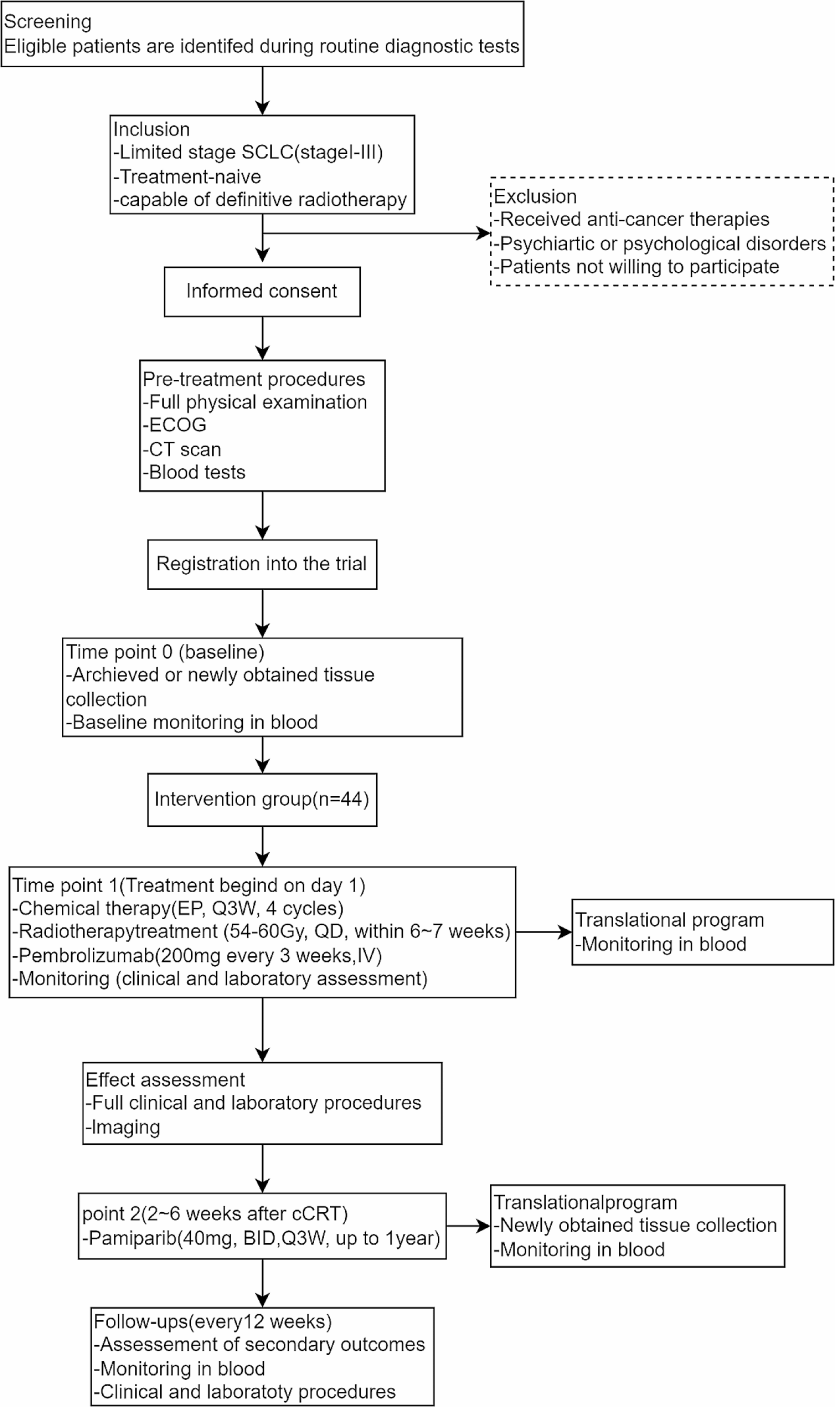
Flowchart of the study. *ECOG* Eastern Co-operative Oncology Group, *MRI* magnetic resonance imaging, *CT* computed tomograph

This study is a prospective, single-arm, phase II clinical trial, with 1-year PFS as the endpoint, to evaluate the efficacy and associated toxicity of Pamiparib as single-agent consolidation treatment in patients with LS-SCLC who have not progressed following platinum-based concurrent chemoradiotherapy (cCRT). This protocol was approved by the ethics committee of Fudan University Shanghai Cancer Centre (Ethics number: 2206255-15) and registered on clinicaltrials.gov as NCT05483543. A flowchart of the study design is depicted in Fig. 1.

### Objectives

#### Study endpoints

*Primary endpoint*

- 1-year progression free survival rate, the percentage of patients who did not experience disease progression as defined by response evaluation criteria in solid tumors (RECIST) v1.1 or death due to any cause within 1 year from the date cCRT treatment ended.

*Secondary endpoints*

- Progression-free survival according to RECIST v1.1 from date of cCRT treatment ended until the date of first documented progression or date of death from any cause, whichever came first, assessed up to 24 months. For patients whose disease did not progress, PFS was evaluated by censoring patients at their most recent imaging.
- Overall survival from date of cCRT treatment ended until the date of end of treatment visit or date of death from any cause, whichever came first, assessed up to 24 months. Participants still alive at the time of data analysis will be censored at the date of last follow-up.
- Overall Response Rate quantifies the proportion of patients with measurable baseline lesions achieving a complete response or partial response as per RECIST 1.1 criteria.
- Duration of Response measures the time from the first documented objective response according to RECIST 1.1 criteria to disease progression or death from any cause, including only patients achieving objective response.
- Distant Metastasis-Free Survival is the interval from the completion of cCRT until the first occurrence of distant metastasis or death from any cause, incorporating metastases such as separate tumor nodules in contralateral lung lobes, tumor with nodules or malignant effusions involving the pleura or pericardium, or extrathoracic metastases.
- The level of AEs defined by NCI-CTCAE v5.0. Safety assessments will be assessed and documented after initiation of study drug, regardless of relationship to study drug.

#### Exploratory purpose

- Univariate and multivariate analysis of the correlation between the molecular type, immune microenvironment, gene expression profile, tumor mutation burden, ctDNA clearance, etc. of LS-SCLC patients and the efficacy of PARPi consolidation therapy;
- Construct a biomarker prediction model for PARPi consolidation therapy-related adverse reactions based on the molecular characteristics of small cell lung cancer patients;
- The correlation between the changes of DNA damage repair genes before and after concurrent chemoradiotherapy and the curative effect of concurrent chemoradiotherapy.

#### Eligibility criteria

*Inclusion criteria*

- The patient or a legally authorized representative must provide study-specific informed consent prior to study entry, had good compliance, and cooperated with the follow-up.
- Age at least 18 years.
- Pathologically (histologically or cytologically) proven diagnosis of limited stage small cell lung cancer (Stage Tx, T1-T4, N0-3, M0, American Joint Committee on Cancer staging, 8th edition), within 60 days prior to registration.
- Patients must have measurable disease (per Response Evaluation Criteria in Solid Tumors [RECIST], version 1.1) prior to the required cycle of cCRT.
- Patients must be free of disease progression and not be able to receive other antitumor therapy within 6 weeks of completion of cCRT.
- Patients must submit archived or freshly biopsied tumor tissue (formalin-fixed, paraffin-embedded tissue block or approximately 15 unstained sections [must have > 8 sections]) along with the relevant pathology report.
- Eastern Cooperative Oncology Group (ECOG) performance status of 0–2 within 30 days prior to registration.
- Patient life expectancy must be > 12 weeks.

*Exclusion criteria*

- Mixed SCLC or NSCLC confirmed by histology.
- Previous tumor resection for LS-SCLC.
- Any patient treatable by surgery or stereotactic body radiation therapy/stereotactic ablative radiation therapy should be excluded.
- Expected to receive any other form of anti-tumor therapy during the study period.
- Previous treatment with PARP inhibitor drugs.
- Any active malignancy within 2 years prior to enrollment, excluding the specific cancers being studied in this study and locally recurrent cancers that have been cured (e.g., resected basal or squamous cell skin cancer, superficial bladder cancer, cervical cancer carcinoma in situ or carcinoma in situ of the breast).
- Women who are pregnant, breastfeeding, or planning to become pregnant during the study.
- Concurrent participation in another therapeutic clinical trial.

### Intervention

Eligible patients will receive Pamiparib 40 mg twice daily every 3 weeks after cCRT up to 1 year or disease progression according to RECIST v1.1 occur.

#### Chemotherapy

The chemotherapy regimen was cisplatin 25 mg/m^2^ on day 1 combined with etoposide 100 mg/m^2^ on days 1, 2 and 3 for 4 cycles. Dosage adjustments based on renal, hematologic, or other toxicity are permitted after the first cycle. If patients have contraindications or intolerance to cisplatin, carboplatin and etoposide will be used as alternative chemotherapy regimens. Carboplatin should be infused intravenously over 15 to 60 min on day 1 of each cycle at a dose of area under the plasma or serum concentration-time curve of 5 (AUC 5) every 3 weeks for 4 cycles and should be administered on Etoposide 100 mg/m^2^ was administered on days 1, 2, and 3 of each cycle for a total of 4 cycles.

#### Radiotherapy

RT should be started after the end of the second cycle of chemotherapy. If this is not possible, RT should be started no later than the end of the third cycle of chemotherapy.

Before enrolling any patient into this study, a radiation oncologist will evaluate a chest CT scan or MRI to ensure that the treatment volume is unlikely to significantly exceed the prescribed normal tissue tolerance and that the patient is receiving RT within the dose range allowed by the protocol. feasible. Patients were excluded if their radiotherapy plan was likely to result in > 20 Gy (V20) radiation doses to greater than 38% of the whole lung volume.

All patients will be treated with standardized three-dimensional conformal radiotherapy techniques or intensity-modulated radiotherapy or volumetric rotational intensity-modulated radiotherapy on a linear accelerator with beam energy > 6 MV. The total dose of RT was 54 to 60 Gy once daily for 6 to 7 weeks.

Since SCLC is sensitive to chemotherapy and RT may not be started until cycle 2 or later (at the latest on day 1 of cycle 3), tumor shrinkage may occur from diagnosis to RT. The recommended field includes the entire affected lymph node region at the time of diagnosis, but the radiotherapy profile of the primary lung tumor can be delineated by the post-chemotherapy volume.

PCI is at the discretion of the investigator. The preferred total dose for whole-brain PCI is 25 Gy delivered in 10 divided doses, once daily.

#### Pamiparib

In patients who have not progressed after concurrent chemoradiotherapy, Pamiparib will start treatment within 2–6 weeks after concurrent chemoradiotherapy as determined by the investigator. The recommended clinical current routine use dose is 40 mg (2 capsules), 2 times a day, orally; Continue dosing for up to 12 months or until RECIST version 1.1-defined disease progression, unacceptable toxicity, death, or another discontinuation criterion, whichever occurs first.

### Sample size calculation

This study will conduct a single-arm, phase II clinical trial in patients with LS-SCLC who have not progressed after platinum-based cCRT. Selected patients will receive consolidation therapy with pamiparib monotherapy, with 1-year PFS as the end point of the study to evaluate the efficacy and related toxicity of pamiparib consolidation therapy. In previous studies of cCRT in LS-SCLC, the 1-year PFS rate was only 43.8–52.8% [31–33]; patients received tarazopanib 1.0 mg/d, and the median PFS was 11.1 weeks [34]. We presupposed that the study protocol would increase PFS from 45 to 60%, α = 0.1, and 39 patients were expected to be enrolled, and 10% dropout was considered, and a total of 44 patients were enrolled. The final analysis of PFS was estimated to be approximately 12 months after the last patient was enrolled to ensure mature PFS data and long-term safety follow-up.

## Discussion

PARPi as a single agent, or in combination with other antitumor regimens for SCLC, has been evaluated mainly in the extensive stage. Single-agent niraparib maintenance therapy demonstrated a moderate improvement in the PFS of platinum-responsive ES-SCLC patients [35]. In a phase II trail, the combination therapy with durvalumab and olaparib revealed an ORR of 10.5% and clinical benefits in 21% of patients in 20 recurrent SCLC patients [36]. The potential use of PARPi as maintenance therapy in SCLC has been also evaluated. A randomized phase III trial evaluating olaparib maintenance demonstrated a slight improvement of median PFS for chemotherapy-sensitive SCLC patients, although OS showed no benefit [37]. Notably, this study did not specifically select for SCLC patients, with only 30% of the experimental group representing limited-stage disease, and a mere 5–8% completing concurrent radiotherapy. Based on these evidences, we conducted a phase II trial to investigate the efficacy of pamiparib, with 16-fold higher PARP1 trapping capability compared to olaparib demonstrated in an in vivo HR-deficient xenograft model [25], in consolidation therapy for LS-SCLC. This study holds promising prospects for achieving favorable outcomes.

The Pamiparib we have chosen has three main advantages. Firstly, compared to the previous PARP inhibitor, Pamiparib has stronger drug efficacy and higher PARP1 trapping ability. Secondly, extensive preclinical investigations have demonstrated the radiosensitizing properties of PARP inhibitors. These inhibitors exhibit various effects, including the direct inhibition of repairing single-strand breaks (SSBs) caused by exposure to ionizing radiation (IR), delayed base excision repair (BER), facilitation and disruption of replication forks, ultimately leading to increased formation of DSBs following radiotherapy [38, 39]. PARPi also improve tumor hypoxia thereby enhance its sensitivity to IR [40]. Furthermore, studies indicate that PARP inhibitors can enhance radiotherapy-induced ferroptosis and trigger antitumor immune responses through the cGAS signaling pathway [41]. Thirdly, Pamiparib demonstrates exceptional penetration capabilities across the BBB. Research has confirmed its strong BBB permeability, thereby enhancing the effectiveness of TMZ in SCLC brain metastasis and glioblastoma models [25]. Preclinical studies have revealed that Pamiparib more effectively enhances radiotherapy compared to Veliparib in high-grade glioblastoma models, highlighting the combined benefits of radiosensitization and BBB penetration conferred by Pamiparib [42]. The brain is the most common site of distant failure in patients diagnosed with LS-SCLC who undergo cCRT. Historical studies have reported brain recurrence rates ranging from 20 to 29% [43–45]. Pamiparib has the potential to reduce the risk of brain metastases and offer corresponding survival benefits as a consolidation therapy following cCRT in LS-SCLC patients.

In conclusion, this is the first study to evaluate the efficacy and safety of pamiparib as consolidation treatment after concurrent chemoradiotherapy in patients with LS-SCLC. The results of our clinical trial will provide valuable information for developing new treatment strategies for these patients.

## Data Availability

The datasets used and/or analyzed in the current study are available from the corresponding author on reasonable request.

## Abbreviations

LS-SCLC: Limited-staged small cell lung cancer
PARP: Poly(ADP-ribose) polymerases
cCRT: Concurrent chemoradiotherapy
HR: Homologous recombination
ROS: Reactive oxygen species
CNS: Central nervous system
BBB: Blood-brain barrier
RECIST: Response evaluation criteria in solid tumors
PFS: Progression free survival
mOS: Median overall survival
BER: Base excision repair
SSB: Single-strand breaks
IR: Ionizing radiation
DSB: Double-strand breaks
